# Investigation of Polymorphisms Induced by the Solo Long Terminal Repeats (Solo-LTRs) in Porcine Endogenous Retroviruses (ERVs)

**DOI:** 10.3390/v16111801

**Published:** 2024-11-20

**Authors:** Cai Chen, Zhanyu Du, Yao Zheng, Hong Chen, Ahmed A. Saleh, Naisu Yang, Mengli Wang, Phiri Azele, Xiaoyan Wang, Chengyi Song

**Affiliations:** 1 College of Animal Science and Technology, Yangzhou University, Yangzhou 225009, China; 007302@yzu.edu.cn (C.C.); mz120180996@yzu.edu.cn (Y.Z.); mx120230878@stu.yzu.edu.cn (H.C.); elemlak1339@gmail.com (A.A.S.); dx120180101@yzu.edu.cn (N.Y.); wangmengli24021@outlook.com (M.W.); wxyan@yzu.edu.cn (X.W.); 2 International Joint Research Laboratory, Universities of Jiangsu Province of China for Domestic Animal Germplasm Resources and Genetic Improvement, Yangzhou 225009, China; 3 College of Grassland Resources, Institute of Qinghai-Tibetan Plateau, Southwest Minzu University, Chengdu 610225, China; yuzhan.du@outlook.com; 4 Animal and Fish Production Department, Faculty of Agriculture (Al-Shatby), Alexandria University, Alexandria City 11865, Egypt; 5 Ministry of Fisheries and Livestock, Animal Science and Technology, Zambia Institute of Animal Health, Mazabuka 670237, Zambia; phiriazele86@outlook.com

**Keywords:** solo-LTR, polymorphic sites, population genetics, genomic variation

## Abstract

Homologous recombination events take place between the 5′ and 3′ long terminal repeats (LTRs) of ERVs, resulting in the generation of solo-LTR, which can cause solo-LTR-associated polymorphism across different genomes. In the current study, specific criteria were established for the filtration of solo-LTRs, resulting in an average of 5630 solo-LTRs being identified in 21 genomes. Subsequently, a protocol was developed for detecting solo-LTR polymorphisms in the pig genomes, resulting in the discovery of 927 predicted solo-LTR polymorphic sites. Following verification and filtration processes, 603 highly reliable solo-LTR polymorphic sites were retained, involving 446 solo-LTR presence sites (solo-LTR^+^) and 157 solo-LTR absence sites (solo-LTR^−^) relative to the reference genome. Intersection analysis with gene/functional regions revealed that 248 solo-LTR^−^ sites and 23 solo-LTR^+^ sites overlapped with genes or were in the vicinity of genes or functional regions, impacting a diverse range of gene structures. Moreover, through the utilization of 156 solo-LTR polymorphic sites for population genetic analysis, it was observed that these solo-LTR loci effectively clustered various breeds together, aligning with expectations and underscoring their practical utility. This study successfully established a methodology for detecting solo-LTR polymorphic sites. By applying these methods, a total of 603 high-reliability solo-LTR polymorphic sites were pinpointed, with nearly half of them being linked to genes or functional regions.

## 1. Introduction

Previous studies have shown that LTR retrotransposons, which include long terminal repeat elements at both ends and protein-coding sequences internally, constitute a significant portion of the pig genome, comprising 7.56% of its content. LTR retrotransposons are generally classified into five superfamilies: Copia, Gypsy, BEL, DIRS, and endogenous retroviruses (ERVs), with ERVs being the most prominent type, accounting for 7.43% of the pig genome [1]. Most ERVs in the pig genome have decayed, and only about 250 candidates retain intact reverse transcription (RT) regions [1,2].

Genomic DNA analysis has identified four possible states of endogenous retroviruses (ERVs) within a host genome [1,3,4]: (1) A complete ERV, also known as a modern ERV, which has recently invaded the genome and has maintained its intact structure; (2) A truncated ERV, which retains recognizable ERV structures but is incomplete due to accumulated mutations; (3) A solo-long terminal repeat (solo-LTR), characterized by the presence of a single LTR due to homologous recombination, where one LTR and the internal coding regions have been excised; (4) An empty site, where no ERV integration has occurred (Figure S1).

Solo-LTRs are prevalent in humans and mice and can result in presence-absence polymorphism at specific sites [5,6,7]. Their de novo identification poses challenges and typically requires comparison with ERV LTR sequences [8]. In a previous analysis of LTR retrotransposons [1], we observed a substantial number of solo-LTRs in the pig genome. These solo-LTRs exhibit insertional polymorphism among different individuals, indicating their potential as valuable material for developing molecular markers. Given the inherent presence of promoters, enhancers, and transcription factor binding sites within LTR elements [9,10], these sequences have the capability to modulate gene expression [1,11]. Research on the human beta-globin gene cluster has revealed that the upstream ERV-9 LTR can recruit transcription factors to the downstream globin gene promoter via long-range chromatin interactions and the expression of long non-coding RNA [12]. *RLTR13D5*, the sequence of which originally derives from a long terminal repeat (LTR) segment of an ERV, contributes hundreds of mouse-specific H3K4me1/H3K27ac-defined enhancers and is capable of driving gene expression in rat placental cells [13]

Research on solo-LTRs in livestock genomes is limited. In this study, solo-LTRs in the genomes of pigs were systematically identified. The polymorphisms associated with solo-LTRs were meticulously predicted across 21 genomes and subsequently validated through PCR. A comprehensive population genetic analysis was then conducted using the identified solo-LTR polymorphic sites, shedding light on the genomic landscape of solo-LTR diversity and evolution in the porcine population. These analyses significantly enhance our understanding of the genetic modifications induced by solo-LTR in the pig genome. Furthermore, this investigation contributes to advancing our knowledge of the mechanisms regulating gene expression and the diverse phenotypic traits observed in pigs.

## 2. Materials and Methods

### 2.1. Genomes and Gene Annotation Sources

The pig reference genome (Sscrofa11.1) and 20 assembled genomes (Table S1) from the NCBI Genome database were utilized for the comprehensive mining of solo-LTR polymorphic sites. The study included a total of 21 pig genomes, representing a diverse range of pig breeds. Seven genomes were sourced from commercial breeds bred in Europe or America, including GCA_000003025.6 (Duroc), GCA_001700135.1 (Large White), GCA_001700165.1 (Hampshire), GCA_001700215.1 (Landrace), GCA_001700255.1 (Pietrain), GCA_001700575.1 (Berkshire), and GCA_015776825.1 (Duroc). One genome was obtained from the Large White_Landrace_Duroc hybrid pig GCA_002844635.1 (Cross-bred). One genome from the Ellegaard Gottingen minipig from Germany, one genome from the Nero Siciliano pig from Italy, and one genome from the PK15 cell line. Additionally, eight genomes originated from Chinese native pig breeds, including GCA_000325925.2 (Wuzhishan), GCA_000472085.2 (Tibetan), GCA_001700155.1 (Rongchang), GCA_001700195.1 (Meishan), GCA_001700235.1 (Bamei), GCA_001700295.1 (Jinhua), GCA_007644095.1 (Bama), and GCA_017957985.1 (Meishan). Detailed information about the genomes can be obtained from https://www.ncbi.nlm.nih.gov/datasets/genome/?taxon=9823 (accessed on 16 February 2023) and Table S1. Those assembled genomes, obtained through next-generation/third-generation sequencing technologies, are referred to as non-reference genomes in this study. The gene annotation files employed here were consistent with previous studies [14]. Specifically, the lncRNA gene annotation file was sourced from the NONCODE database (http://www.noncode.org/download.php, (accessed on 16 May 2020)). Information regarding protein-coding genes and the details of exons and introns within them were extracted from the Sscrofa11.1 annotation on the NCBI database (https://ftp.ncbi.nlm.nih.gov/genomes/all/annotation_releases/9823/106, (accessed on 16 May 2020)). Additionally, data on enhancer regions and enhancer RNA (eRNA) regions were obtained from Pig-eRNAdb [15].

### 2.2. Pig Custom Repeat Library of ERVs

In previous studies, a thorough re-evaluation of retrotransposons in the pig genome led to the discovery of new retrotransposons. Subsequently, these findings were integrated with data from the Repbase library to craft a pig custom repeat library tailored for the pig genome, which contains 1174 sequences of which 295 belong to ERV, and the sequences of ERV were splinted to internal parts and LTR element parts [1]. Finally, 32 LTR element consensus sequences (1 Gypsy element and 31 ERV elements) with an average length of 416 bp (ranging from 104 to 785 bp) were selected for solo-LTR polymorphic sites mining (Table S2 and Supplementary File S1: LTR-element-sequence.fas).

### 2.3. Pig Genomes Annotation with RepeatMasker

The pig reference genome along with 20 non-reference genomes underwent annotation using RepeatMasker [16] (version 4.0.9, -nolow) with the pig custom repeat library [1]. The K divergence of these 32 LTR elements was then calculated utilizing the calc-DivergenceFromAlign.pl tool within the RepeatMasker program.

### 2.4. Solo-LTR Identification

Building upon the repeat annotation outcomes from the pig reference genome, the identification of solo-LTRs across the entire genome was conducted through a series of steps: (a) Extraction all LTR retrotransposons into positive and negative strands based on annotation details. (b) Determination of solo-LTRs based on specific criteria, including (1) retaining LTR elements with a minimum length of 50 bp and (2) excluding LTR elements with annotated information on LTR retrotransposons within 500 bp upstream and downstream. (c) Consolidation of solo-LTR data from both strands and calculating the total number of solo-LTRs. Unique identifiers were assigned, and genomic locations were documented. (d) Application of the same methodology used for the reference genome to identify solo-LTRs in the non-reference genomes.

### 2.5. Solo-LTR Polymorphic Sites Mining

A comprehensive protocol for exploring solo-LTR polymorphic sites throughout 21 pig genomes was devised, comprising 3 primary steps.

Step 1. Mapping to the Reference Genome

(a) The bedtools flank and bedtools getfasta tools were utilized to extract the nucleotide sequences of the 200 bp upstream flanking regions of solo-LTRs in the non-reference genome. (b) The blat tool was utilized with specific parameters (-minIdentity 90; -minScore 180) to align the 200 bp upstream sequences with the reference genome. Subsequently, a filtration criterion was applied, ensuring that (1) mapping results had lengths falling within the 180~220 bp range and (2) each solo-LTR mapped uniquely to a single genomic position, excluding those mapping to multiple positions. (c) In cases where solo-LTRs failed to map to the reference genome using the upstream 200 bp flanking sequences, mapping was attempted with the downstream 200 bp flanking sequences, and the above filtration criterion was also applied. (d) Subsequently, the mapping outcomes derived from procedures b and c, which employed either 200 bp upstream or downstream flanking sequences, were integrated to accurately pinpoint the precise positional details of solo-LTR insertions within non-reference genomes that had been successfully aligned to the reference genome.

Step 2: Cross-Comparison

For solo-LTRs from non-reference genomes that successfully aligned with the reference genome, the bedtools window tool with a parameter of -w 50 was used to determine overlaps with the coordinates of all LTR elements in the reference genome, not solely solo-LTRs. Consequently, solo-LTRs in non-reference genomes that did not align with any LTR element in the reference genome were identified as deletion-type solo-LTR polymorphic sites (solo-LTR^−^) originating from the non-reference genome. Blast analysis was then performed. The bedtools slop and bedtools getfasta tools extended each non-reference genome-derived solo-LTR by 200 bp on both sides to extract sequences from the respective non-reference genome. Subsequently, a blast utilizing parameters (-task megablast, -evalue 1.0 × 10^−5^, -max_target_seqs 1, -max_hsps 1) was carried out against the reference genome to pinpoint those solo-LTRs from the non-reference genome exhibiting polymorphisms. Finally, the solo-LTR polymorphic sites remaining from all non-reference genomes were merged using bedtools merge (-s -d 10), and redundancies were eliminated to obtain unique non-reference genome-derived solo-LTR polymorphic sites.

In the reference genome, 200 bp flanking sequences on both sides of each solo-LTR along with their sequences were extracted using the bedtools slop and bedtools getfasta tools. Then, blast was performed against the non-reference genomes to pinpoint those solo-LTRs from the reference genome exhibiting polymorphisms. The refined outcomes were consolidated, and the distinct solo-LTR polymorphic sites originating from the reference genome were identified.

Step 3: PCR validation for the solo-LTR polymorphic sites

PCR validation was conducted for the solo-LTR polymorphic sites, the primer pairs were designed based on the up- and downstream flanking regions of the selected solo-LTR polymorphic sites (primers listed in the Supplementary File). Ear and blood samples were collected from 12 pig breeds, namely Duroc, Large White, Landrace, Meishan, Mi, Sushan, Bamei, Ningxiang, Bama, Banna, Wuzhishan, and Tibetan pigs. Three individuals from each breed were selected, and DNA was extracted using TianGen’s DNA extraction kit. Equal pooling of DNA samples from the same breed yielded 12 pools. PCR was performed with specific primers for each solo-LTR along with their corresponding Tm. The resulting products underwent agarose gel electrophoresis using 1 × TAE buffer and DL2000 as a molecular weight marker. Following staining with a nucleic acid dye reagent for 15 min, gel images were captured and analyzed to evaluate the electrophoretic outcomes.

### 2.6. Annotation of Solo-LTR Polymorphic Sites

Initially, an analysis was conducted to evaluate the distribution of solo-LTR polymorphic sites across the genome and investigate their correlation with genes. This involved assessing the quantity and density statistics of solo-LTR polymorphic sites on each chromosome, followed by a comprehensive exploration of the relationships between solo-LTR polymorphic sites and genes, including their functional regions.

### 2.7. Utilization of Solo-LTR Polymorphic Sites for Population Analysis

The 156 solo-LTR polymorphic sites showing presence state in 5–16 genomes, classified as common occurrence sites, were utilized for population analysis utilizing the Principal Component and Heat Map with Dendrogram features in Origin 2024 (Version 2024). Initially, we documented the absence/presence status of all common sites across 21 genomes, obtaining the genotyping results. Subsequently, we replaced “absence” with “0” and “presence” with “1”. Then, we imported the data into Origin 2024 software and used its “Principal Component” and “Heat Map with Dendrogram” modules for visualization. The data normalization parameter was set to column-wise for pheatmap analysis, while all other parameters were left at their default values.

### 2.8. Statistical Tests

Spearman’s correlation analysis was performed for the correlation between the number of solo-LTR polymorphic sites and the size of each chromosome and the correlation between the number of solo-LTRs and the number of LTR elements using SPSS (version 16.0; Chicago, IL, USA).

## 3. Results

### 3.1. A Large Number of Solo-LTRs Were Present in the Pig Genome

**Figure 1:**
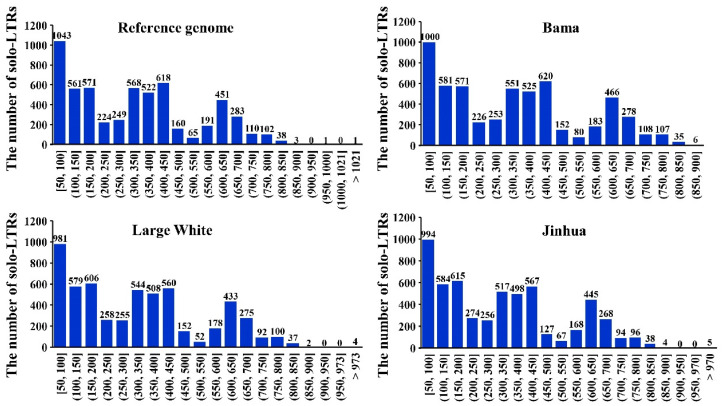
The length distribution of solo-LTRs in pig reference genome, Bama, Large White, and Jinhua genomes. The x-axis represents length intervals, and the y-axis represents the number of solo-LTRs.

By applying the series of criteria introduced in the Materials and Methods, an average of 5630 solo-LTRs were detected in the 21 genomes, with quantities ranging from 3489 in the Ellegaard Gottingen minipig to 6656 in the Cross-bred (Table 1). Our length analysis of solo-LTRs in the reference genome revealed 2175 instances with lengths below 200 bp. Additionally, there were notable distributions in the 300–450 and 600–700 bp intervals, while other lengths were less prevalent. This trend was highly similar in the genomes of Bama, Large White, and Jinhua (Figure 1). A similar length distribution pattern was also observed in the remaining genomes (Figure S2). Mapping these solo-LTRs from non-reference genomes to the reference genome by using their 200 bp flanking regions resulted in an average successful mapping rate of 94.51%, ranging from 81.66% in the Cross-bred to 97.74% in the Ellegaard Gottingen minipig (Table 1). These observations lay a strong foundation for the subsequent identification of solo-LTR polymorphic sites.

### 3.2. 603 Solo-LTR Polymorphic Sites Were Identified Across the Pig Genomes

**Figure 2:**
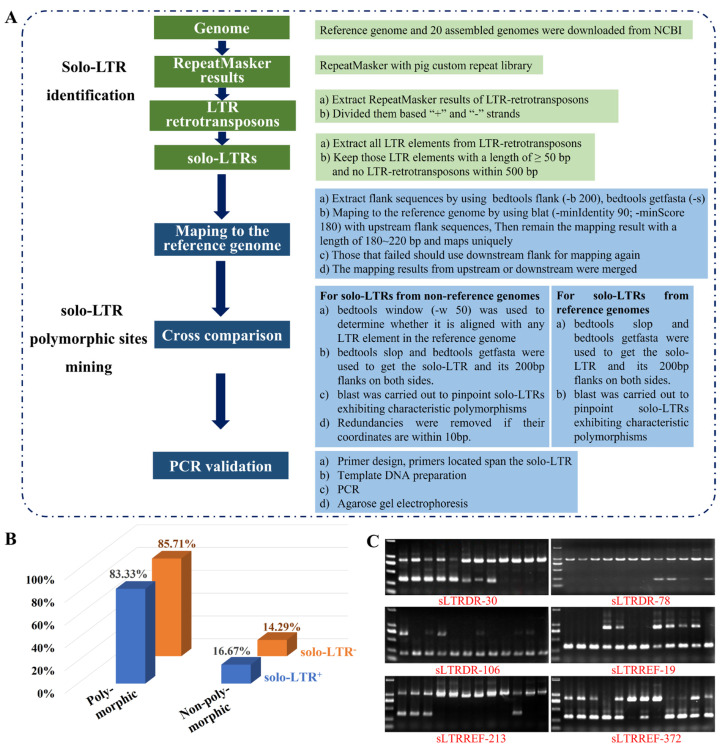
The methodology and PCR verification for the solo-LTR polymorphic sites. (**A**) A genome-wide methodology for detecting solo-LTR polymorphic sites across 21 assembled pig genomes. (**B**) Summary of PCR outcomes for two categories of solo-LTR polymorphic sites: blue indicates solo-LTR^+^, while orange indicates solo-LTR^−^. (**C**) The gel-electrophoresis results for polymorphic sites are presented. Specifically, sLTRDR-30, sLTRDR-78, and sLTRDR-106 were solo-LTR^+^ sites, whereas sLTRREF-19, sLTRREF-213, and sLTRREF-372 were solo-LTR^−^ sites. For each lane, the larger band represents that the solo-LTR is present, while the small band represents that the solo-LTR is absent. Lane order: DL2000 marker, Duroc, Large White, Landrace, Bamei, Ningxiang, Bama, Wuzhishan, Meishan, Mi, Sushan, Tibetan, and Banna pigs.

A genome-wide mining protocol for detecting solo-LTR polymorphic sites across 21 assembled pig genomes (20 non-reference and 1 reference) was established and is detailed in the methodology section (Figure 2A). A site was designated as solo-LTR^+^ if a solo-LTR was present at a specific position in the reference genome but absent in the equivalent position in non-reference genomes; conversely, the opposite situation was designated as solo-LTR^−^. In total, 927 solo-LTR polymorphic sites were predicted based on the genome-wide analysis, with 481 sites identified as solo-LTR^+^ and 446 as solo-LTR^−^ in the reference genome.

The decreased integrity of the non-reference genome compared to the reference genome could have led to fragment losses, resulting in false positives of solo-LTR polymorphic sites due to differences in sequencing and assembly quality. Combining our expertise in detecting SINE-RIPs [14], we eliminated the solo-LTR^+^ sites that the solo-LTR was just absent in no more than three non-reference genomes. This exclusion step resulted in the elimination of 324 solo-LTR polymorphic sites, resulting in a final count of 603 solo-LTR polymorphic sites (coordinates provided in Supplementary File S2).

To evaluate the polymorphism rate of the retained solo-LTR polymorphic sites across individuals, we randomly selected 30 solo-LTR^+^ and 52 solo-LTR^−^ sites for PCR verification using 12 pooled DNA samples as templates. The findings indicated that, among the solo-LTR^+^ sites, 6 sites were indeterminate, 83.33% (20 out of 24) exhibited polymorphism, and 16.67% (4 out of 24) were monomorphic. Regarding solo-LTR^−^, 10 sites were indeterminate, 85.71% (36 out of 42) were polymorphic, and 14.29% (6 out of 42) were monomorphic (Figure 2B). Overall, above 80% of the solo-LTR polymorphic sites were confirmed by PCR. The electrophoresis results for all examined sites are depicted in Figure 2C, Figures S3 and S4, and the genotyping outcomes are provided in Supplementary File S2.

**Figure 3:**
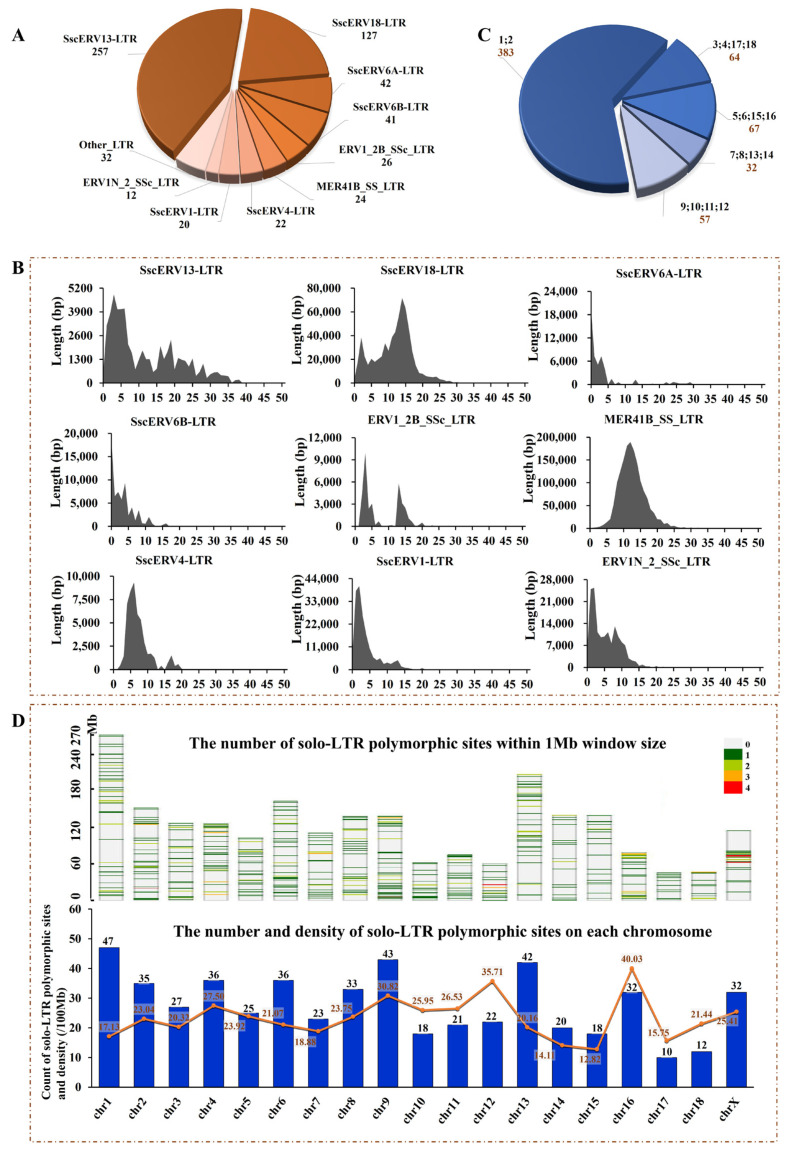
603 solo-LTR polymorphic sites source analysis and frequency and distribution analysis. (**A**) The majority of the solo-LTR polymorphic sites originate from 9 ERV families. (**B**) The divergence of LTR elements derived from 9 prominent ERV sources within the pig genome. The x-axis indicates the K divergence calculated (%) using the calc-DivergenceFromAlign.pl tool within the RepeatMasker program, while the y-axis represents the total number of bases marked as a specific LTR element in the genome, reflecting the content level at the respective divergence. (**C**) Frequency distribution of 603 solo-LTR polymorphic sites across 21 genomes; the number represents the genomes presenting the specific solo-LTR. (**D**) The distribution of 603 solo-LTR polymorphic sites on each chromosome of the pig genome. The above part is the distribution of 603 solo-LTR polymorphic sites on each chromosome. The below is the number of solo-LTR polymorphic sites on each chromosome (blue bar and the numbers at the top) and the density of solo-LTR polymorphic sites per 100 M on each chromosome (orange line and the adjacent numbers).

Among the 32 LTR elements examined, 20 contributed to the total of 603 solo-LTR polymorphic sites. Nine LTR elements—SscERV13-LTR, SscERV18-LTR, SscERV6A-LTR, SscERV6B-LTR, ERV1_2B_SSc-LTR, MER41B_SS-LTR, SscERV4-LTR, SscERV1-LTR, and ERV1N_2_SSc-LTR—were the main contributors, with SscERV13-LTR being the predominant, accounting for 257 solo-LTR polymorphic sites (Figure 3A). Notably, SscERV6A-LTR and SscERV6B-LTR stand out as SscERV6 is the most recent ERV in the pig genome, contributing to 83 solo-LTR polymorphic sites. Analysis of 32 LTR elements in the pig genome indicated that these 9 LTR elements exhibit relatively lower divergence within the pig genome compared to other LTR elements (Figure 3B and Figure S5). Further evaluation of the number of solo-LTR presence sites among these 603 solo-LTR polymorphic sites across 21 genomes revealed that there are 383 sites showing presence state in only 1–2 genomes, regarded as rare occurrence sites, which constituted 63.51% (383 out of 603) of the solo-LTR polymorphic sites. This suggests that most of the solo-LTR polymorphic sites are specific to individual genomes or the species as a whole. Moreover, there are 156 sites showing presence state in 5–16 genomes, classified as common occurrence sites, and 64 sites in 3–4 or 17–18 genomes, which are regarded as median occurrence sites (Figure 3C, Supplementary File S2).

The distribution analysis of solo-LTR polymorphic sites on autosomes and the X chromosome was undertaken, totaling 532 solo-LTR polymorphic sites from 18 autosomes and the X chromosome. Due to the significant presence of Ns on the Y chromosome, it was excluded from the distribution analysis. On chromosomes 2, 9, and 12, there is one cluster each, while on the X chromosome, there are three clusters (Figure 3D). On average, each chromosome contained 28 solo-LTR polymorphic sites, with chromosome 17 having the least at 10, and chromosome 1 the most at 47. Statistical analysis revealed a significant correlation between the number of identified solo-LTR polymorphic sites on each chromosome and the chromosome size (*p* < 0.01). The density analysis revealed an average of 23.39 solo-LTR polymorphic sites per 100 Mb on each chromosome, with chromosome 16 displaying the highest density at 40.03 and chromosome 15 the lowest at 12.82. Notably, despite chromosome 1 having the largest number of sites, its density was 17.13, in contrast to chromosome 17 with fewer sites but a density of 15.75 (Figure 3D). These outcomes indicate a relatively even distribution of solo-LTR polymorphic sites across the chromosomes.

### 3.3. About 45% of Solo-LTR Polymorphic Sites Were Overlapping with Genes/Functional Regions

Solo-LTR polymorphic sites in the genome may impact the gene regulation system. To delve deeper into this association, we conducted cross-comparisons between these sites and various genomic regions, including protein-coding genes (genes, introns, exons, upstream and downstream 5 kb flanking regions), lncRNA genes (genes, introns, exons, upstream and downstream 5 kb flanking regions), enhancer regions, and enhancer RNA regions. For solo-LTR^−^ sites, only those intersecting with functional regions and genes were taken into account, while for solo-LTR^+^ sites, only those overlapping by more than 50 bp were included in the analysis. The analysis unveiled that 64 sites intersect with 66 lncRNA genes, all positioned within introns. In the proximity of lncRNA genes, 34 solo-LTR polymorphic sites were detected. In the realm of protein-coding genes, 206 sites were identified, comprising 2 within exon regions of distinct genes and 204 in intron regions, potentially affect 837 transcripts of 187 genes, with 49 located in gene-flanking regions. Additionally, 2 sites were found in pig enhancer regions and 33 in enhancer RNA (eRNA) regions. In total, there were 248 solo-LTR^−^ sites and 23 solo-LTR^+^ sites distributed across genes, gene surroundings, and functional regions, influencing a diverse array of gene structures and potentially playing a vital regulatory role in gene expression (Table 2). GO and KEGG analyses were conducted on protein-coding genes containing solo-LTR polymorphic sites and it was found that these genes are mainly associated with signal transduction, such as post synapse, axon, glutamatergic synapse, and GABAergic synapse (Figure S6).

### 3.4. Utility of Solo-LTR Polymorphic Sites for Population Analysis

**Figure 4:**
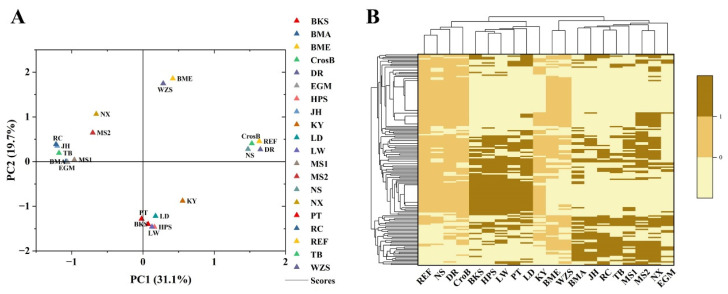
PCA (**A**) and pheatmap (**B**) clustering results with common occurrence sites. (REF: Duroc (Sscrofa11.1); WZS: Wuzhishan; EGM: Ellegaard Gottingen minipig; TB: Tibetan; LW: Large_White; RC: Rongchang; HPS: Hampshire; MS1: Meishan; LD: Landrace; BME: Bamei; PT: Pietrain; JH: Jinhua; BKS: Berkshire; CrosB: Cross-bred (Yorkshire_Landrace_Duroc); NS: Nero Siciliano pig; BMA: Bama miniature; DR: Duroc (Ninghe); MS2: Meishan (Beijing); PK15: PK15 cells; NX: Ningxiang; KY: Kenya domestic pig).

Following the exploration of solo-LTR polymorphic sites throughout diverse pig genomes, the solo-LTR polymorphic sites classified as common occurrence sites were used for principal component analysis (PCA) and pheatmap analysis, which were based on the presence/absence status of each site. The PCA results based on breed (Figure 4A) unveiled four distinct clusters within the genomes. The primary cluster encompassed Chinese native pigs, Wuzishan, Bamei, Meishan, Rongchang, Bama, and Ningxiang, Tibetan and Jinhua pigs, and Gottingen minipigs; the second group comprised two Duroc, a crossbred and a Nero Siciliano pig; whereas Berkshire, Hampshire, Landrace, Pietrain, Large White, and Kenya constituted a separate group. The pheatmap representation categorized the genomes of 18 pig breeds into 5 distinct categories (Figure 4B), closely aligned with the PCA classifications.

## 4. Discussion

Solo-LTRs are standalone elements sharing the structure of the 5’LTR or 3’LTR of intact LTR retrotransposons [17]. Chen et al. detected 86 breed-common PERV insertion polymorphism sites in pigs; 24 were caused by solo-LTRs [18]. In humans, solo-LTRs make up a large majority of the HERVs, and many are involved in various biological processes by acting as promoters/enhancers, which was reviewed in [19,20], with HERV-H contributing about 1000 solo-LTR copies [21,22]. Ji et al. identified 1716 and 2144 solo LTRs in chicken and zebra finch assemblies [23]. These studies highlight the prevalence and significance of solo-LTRs. In the current study, we investigated the 32 LTR elements from the pig genome transposon database established in previous research [1], revealing variations in length and sequence among different LTR elements. On average, 5619 solo-LTRs were detected in the genomes of 21 pig breeds, with 927 predicted solo-LTR polymorphic sites identified. Solo-LTRs were most frequently found at approximately 5000 copies in most genomes but exceeding 6600 in the cross-bred genome, which is likely due to increased recombination events resulting from hybridization. The fact that so many solo-LTRs can exist in the genome might be the reason that most full-length ERV insertions are long (7–10 kb) and not tolerated by host genomes; the full-length ERV insertions will decrease with the adaptation selection in nature, while a short solo-LTR is more tolerable for the host genome. For the length of solo-LTRs, we observed the substantial presence of copies smaller than 200 bp in the pig genome, differing from the length of the LTR consensus sequences. This indicates that solo-LTRs continue to undergo distinct evolutionary events after their generation, with the genome showing a preference for retaining smaller functional segments. In this study, 32 LTR elements were used for solo-LTR mining. Of these, 1 is from the Gypsy element, and 31 are from ERV elements. Thirteen ERVs were obtained from Repbase and were unclassified. Eighteen ERVs, which contain RT domains, were identified. These were classified into spuma, beta, and gamma retroviruses with 1, 4, and 13, respectively [1]. The commonly studied PERV-A and PERV-C, belonging to the SscERV6A subfamily, and PERV-B, belonging to the SscERV6B subfamily, were classified as gamma retroviruses [24,25,26]. We found that the identified solo-LTR polymorphic sites primarily originated from nine LTR elements: SscERV13, SscERV18, SscERV6A, SscERV6B, ERV1_2B_SSc, MER41B_SS, SscERV4, SscERV1, and ERV1N_2_SSc. Five of these (SscERV1, SscERV6A, SscERV6B, SscERV4, and SscERV13) are part of the defined gamma retroviruses, which signify very recent invasions in the genome [1].

Building on our previous research on SINE retrotransposon insertion polymorphisms (SINE-RIPs) [14], we excluded 324 genotypes that were predicted to occur in only 1–3 genomes out of the total 927 solo-LTR polymorphic sites. As a result, the current polymorphism detection rate was approximately 80%, akin to the SINE-RIP polymorphism rate. Some solo-LTR polymorphic sites exhibited poor amplification, a phenomenon also observed in PCR evaluations of SINE-RIPs. These sites may reside in complex structural regions, such as repeat regions, resulting in suboptimal amplification and difficulties in accurately determining genotypes.

Furthermore, LTR retrotransposons and their derived sequences are often encountered in enhancers and other regulatory regions [27,28], repressors [29], or promoters [30] of downstream genes, effectively modulating their expression. Previous studies have highlighted the robust promoter activity of young ERV (ERV6A, 6B) LTR elements [1], suggesting a significant impact of LTRs on gene expression [31,32]. An analysis was conducted on 603 solo-LTR polymorphic sites using the gene annotation file from the reference genome, revealing that 128 solo-LTR polymorphic sites were located within genes. Notably, the analysis unveiled a substantial number of solo-LTR polymorphic sites within or adjacent to genes, indicating a potential influence on gene regulation. The investigation further suggests that solo-LTR polymorphic sites intersect with enhancer RNA regions, potentially exerting a broader impact on gene expression. This underscores the significant role of LTRs in regulating gene expression.

Population genetic analysis was conducted on 21 genomes using 156 common occurrence sites. It was observed that within the same breed, such as Duroc and Meishan, which each have two genomes, the genomes cluster together. Lean-type commercial pig breeds and Chinese native pigs also tend to cluster together, indicating that solo-LTR polymorphic sites provide valuable resources for the development of molecular markers. However, our analysis was limited to homozygous genotypes and did not consider heterozygous cases, and only 156 common occurrence sites were used. These limitations may affect the accuracy of genotyping results, which could lead to the Nero Siciliano pig clustering with Duroc and cross-bred pigs and the minipigs not clustering well together.

## 5. Conclusions

In the current study, an average of 5630 solo-LTRs were identified in the pig genome, and a mining protocol for detecting solo-LTR polymorphic sites was successfully developed. PCR validation confirmed 603 high-confidence sites, nearly half of which intersect with genes or functional regions. A subsequent population genetic analysis using common occurrence sites effectively classified the genetic profiles of 21 genomes. This investigation may serve as a valuable guide for future studies in the same field, providing abundant resources for developing molecular markers and insights into the genetic diversity and evolution of the pig genome.

## Figures and Tables

**Table 1:** The detailed number of solo-LTR in 21 pig genomes.

Number	Genome Name	No. of Solo-LTR	No. of Solo-LTR Mapped to Ref-Genome	Successfully Mapped Ratio (%)
1	Sscrofa11.1	5761	——	——
2	Meishan (Beijing)	5784	5304	91.70
3	Meishan	5624	5459	97.07
4	Bama	5742	5389	93.85
5	Bamei	5594	5439	97.23
6	Berkshire	5544	5389	97.20
7	Cross-bred	6656	5435	81.66
8	Ellegaard Gottingen minipig	3489	3410	97.74
9	Hampshire	5631	5481	97.34
10	Jinhua	5617	5461	97.22
11	Landrace	5606	5461	97.41
12	LargeWhite	5616	5473	97.45
13	Pietrain	5615	5460	97.24
14	Rongchang	5641	5488	97.29
15	Tibetan	5558	5424	97.59
16	Wuzhishan	5855	5611	95.83
17	Duroc (Ninghe)	5763	5410	93.87
18	Kenya domestic pig	5446	5317	97.63
19	Ningxiang	5560	5292	95.18
20	Nero Siciliano pig	5616	5366	95.55
21	PK15 cells	6514	5347	82.08
	Average	5630	5321	94.51

**Table 2:** The overlap results of solo-LTR polymorphic sites with gene or functional regions.

Gene/Functional Region	No. of Solo-LTR			No. of Gene/Transcript
	Solo-LTR^−^	Solo-LTR^+^	Total	Gene/Transcript
lncRNA gene	52	12	64	66
lncRNA gene exon	0	0	0	0
lncRNA intron	52	12	64	64/110
lncRNA 5’flank 5 kb	13	4	17	-
lncRNA 3’flank 5 kb	13	4	17	-
protein coding gene	180	26	206	189
protein coding gene exon	1	1	2	2/2
protein coding intron	179	25	204	187/837
protein coding 5’flank 5 kb	10	10	20	-
protein coding 3’flank 5 kb	18	11	29	-
eRNA region	27	6	33	10
enhancer region	1	1	2	2
Total	248	23	271	-

Note: The solo-LTR^+^ sites overlap with gene/transcript as more than 50 bp were used for counting.

## Data Availability

All data needed to evaluate the conclusions in this paper are present either in the main text or the Supplementary Materials.

## Supplementary Materials

The following supporting information can be downloaded at: https://www.mdpi.com/article/10.3390/v16111801/s1, Table S1. Genome information used for solo-LTR polymorphic sites identification analysis. Table S2. The length of LTR elements in pig genome. Figure S1. Schematic structures of four possible states of endogenous retroviruses (ERVs) within a host genome. Figure S2. The length distribution of solo-LTRs in the left 17 pig genomes. Figure S3. Gel electrophoresis results of 30 solo-LTR^+^ sites. Figure S4. Gel electrophoresis results of 52 solo-LTR^−^ sites. Figure S5. The divergence of LTR elements from 23 LTR retrotransposons within the pig genome are examined. Figure S6. GO and KEGG analyses on protein-coding genes containing solo-LTR polymorphic sites. Supplementary File S1: LTR-element-sequence.fas. Supplementary File S2: solo-LTR-information.xlsx.
