# Supplementary material for: Investigation of Polymorphisms Induced by the Solo Long Terminal Repeats (Solo-LTRs) in Porcine Endogenous Retroviruses (ERVs)
Source: Viruses. 2024 Nov 20;16(11):1801. doi: 10.3390/v16111801 (PMC11598996; doi:10.3390/v16111801)
Supplement: Supplementary file 1 [file viruses-16-01801-s001.zip › 3.Additional file 1_(S.Tables)-2.pdf]

Table S1. Genome information used for solo-LTR polymorphic sites identification analysis.

No.	Assembly	Level	Size(Mb)	Breed/Cell type	Abbreviation	Description	Submitter Country
1	GCA_000003025.6	Chromosome	2501.91	Sscrofa11.1	REF	Commercial pig	American
2	GCA_000325925.2	Scaffold	2508.91	Wuzhishan	WZS	Chinese native pig	China
3	GCA_000331475.1	Contig	2358.02	Ellegaard Gottingen minipig	EGM	German minipig	Germany
4	GCA_000472085.2	Scaffold	2437.74	Tibetan	TB	Chinese native pig	China
5	GCA_001700135.1	Scaffold	2457.91	Large_White	LW	Commercial pig	China
6	GCA_001700155.1	Scaffold	2459.03	Rongchang	RC	Chinese native pig	China
7	GCA_001700165.1	Scaffold	2437.11	Hampshire	HPS	Commercial pig	China
8	GCA_001700195.1	Scaffold	2467.5	Meishan	MS1	Chinese native pig	China
9	GCA_001700215.1	Scaffold	2440.98	Landrace	LD	Commercial pig	China
10	GCA_001700235.1	Scaffold	2460.76	Bamei	BME	Chinese native pig	China
11	GCA_001700255.1	Scaffold	2438.32	Pietrain	PT	Commercial pig	China
12	GCA_001700295.1	Scaffold	2453.7	Jinhua	JH	Chinese native pig	China
13	GCA_001700575.1	Scaffold	2434.71	Berkshire	BKS	Commercial pig	China
14	GCA_002844635.1	Chromosome	2755.44	Cross-bred (Yorkshire_Landrace_Duroc)	CrosB	Commercial pig	China
15	GCA_006511355.2	Chromosome	2479.33	Nero Siciliano pig	NS	Italian native pig	Italy
16	GCA_007644095.1	Chromosome	2491.05	Bama	BMA	Chinese nimipig	China
17	GCA_015776825.1	Chromosome	2458.24	Duroc (Ninghe)	DR	Commercial pig	China
18	GCA_017957985.1	Chromosome	2510.04	Meishan (Beijing)	MS2	Chinese native pig	China
19	GCA_018555405.1	Contig	2598.84	PK15 cells	PK15	MIGS Eukaryotic sample from Sus scrofa	Japan
20	GCA_020567905.1	Chromosome	2440.95	Ningxiang	NX	Chinese native pig	
21	GCA_019290145.1	Scaffold	2444.98	Kenya domestic pig	KY	Kenya native pig	

Table S2. The length of LTR elements in pig genome.

No.	Family	Subfamily	Repbase ID	Length
1	ERV1	ERV1_1B_SSc_LTR	ERV1_1B_SSc_LTR	426
2	ERV1	ERV1_2B_SSc_LTR	ERV1_2B_SSc_LTR	742
3	ERV1	ERV1_4B_SSc_LTR	ERV1_4B_SSc_LTR	345
4	ERV1	ERV1N_1_SSc_LTR	ERV1N_1_SSc_LTR	349
5	ERV1	ERV1N_1B_SSc_LTR	ERV1N_1B_SSc_LTR	374
6	ERV1	ERV1N_2_SSc_LTR	ERV1N_2_SSc_LTR	431
7	ERV1	ERV3_1_SSc_LTR	ERV3_1_SSc_LTR	449
8	ERV1	ERV54_EC_LTR	ERV54_EC_LTR	395
9	ERV1	MER41_SS_LTR	MER41_SS_LTR	785
10	ERV1	MER41B_SS_LTR	MER41B_SS_LTR	697
11	ERV1	MER41C_SS_LTR	MER41C_SS_LTR	689
12	ERV2	ERV2N_1_SSc_LTR	ERV2N_1_SSc_LTR	293
13	ERV3	ERV3_16A3_LTR	ERV3_16A3_LTR	422
14	ERVI	SscERV10-LTR	ERVIN-1A2_Sus_LTR	359
15	ERVI	SscERV11-LTR	-	318
16	ERVI	SscERV12-LTR	-	119
17	ERVI	SscERV13-LTR	-	252
18	ERVI	SscERV1-LTR	ERVI-1_SSc-LTR	429
19	ERVI	SscERV2-LTR	ERVI-4_SSc-LTR	347
20	ERVI	SscERV3-LTR	-	448
21	ERVI	SscERV4-LTR	ERVIN-3_SSc-LTR	429
22	ERVI	SscERV5-LTR	-	720
23	ERVI	SscERV6A-LTR	ERVI-2_SSc-LTRXX	702
24	ERVI	SscERV6B-LTR	-	629
25	ERVI	SscERV7-LTR	-	268
26	ERVI	SscERV8-LTR	ERVI-3_SSc-LTR	308
27	ERVI	SscERV9-LTR	-	104
28	ERVII	SscERV16-LTR	-	352
29	ERVII	SscERV17-LTR	-	184
30	ERVII	SscERV18-LTR	-	498
31	ERVIII	SscERV14-LTR	-	281
32	Gypsy	MamGypsy2_LTR	MamGypsy2_LTR	183
