# Supplementary material for: Investigation of Polymorphisms Induced by the Solo Long Terminal Repeats (Solo-LTRs) in Porcine Endogenous Retroviruses (ERVs)
Source: Viruses. 2024 Nov 20;16(11):1801. doi: 10.3390/v16111801 (PMC11598996; doi:10.3390/v16111801)
Supplement: Supplementary file 1 [file viruses-16-01801-s001.zip › 4.Additional file 1_(S.Figures).pdf]

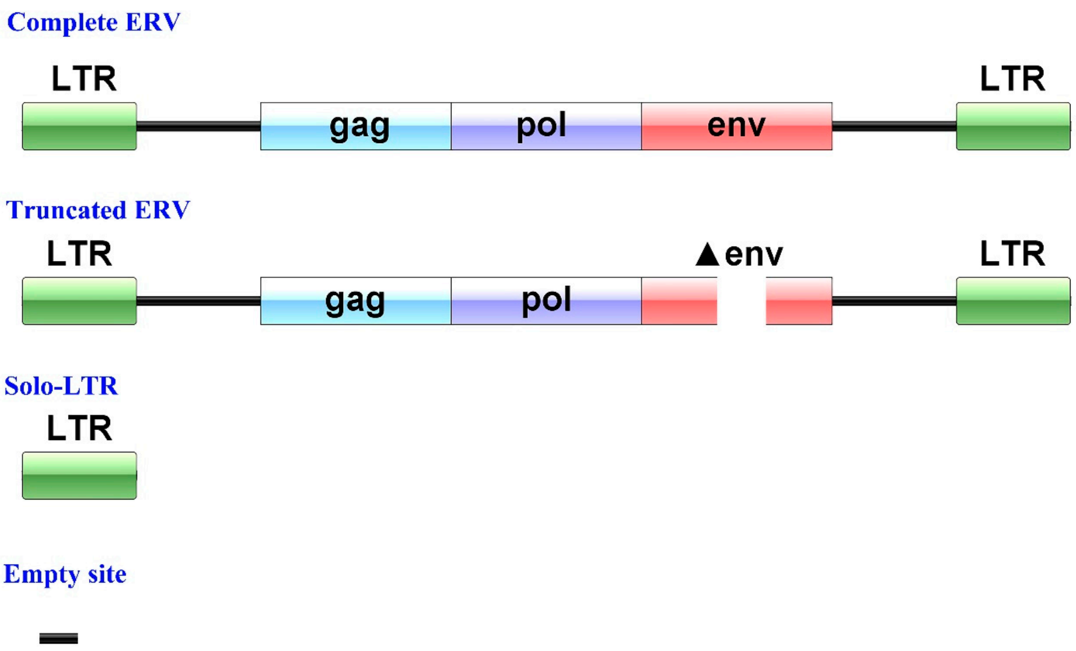

Figure S1. Schematic structures of four possible states of endogenous retroviruses (ERVs) within a host genome .

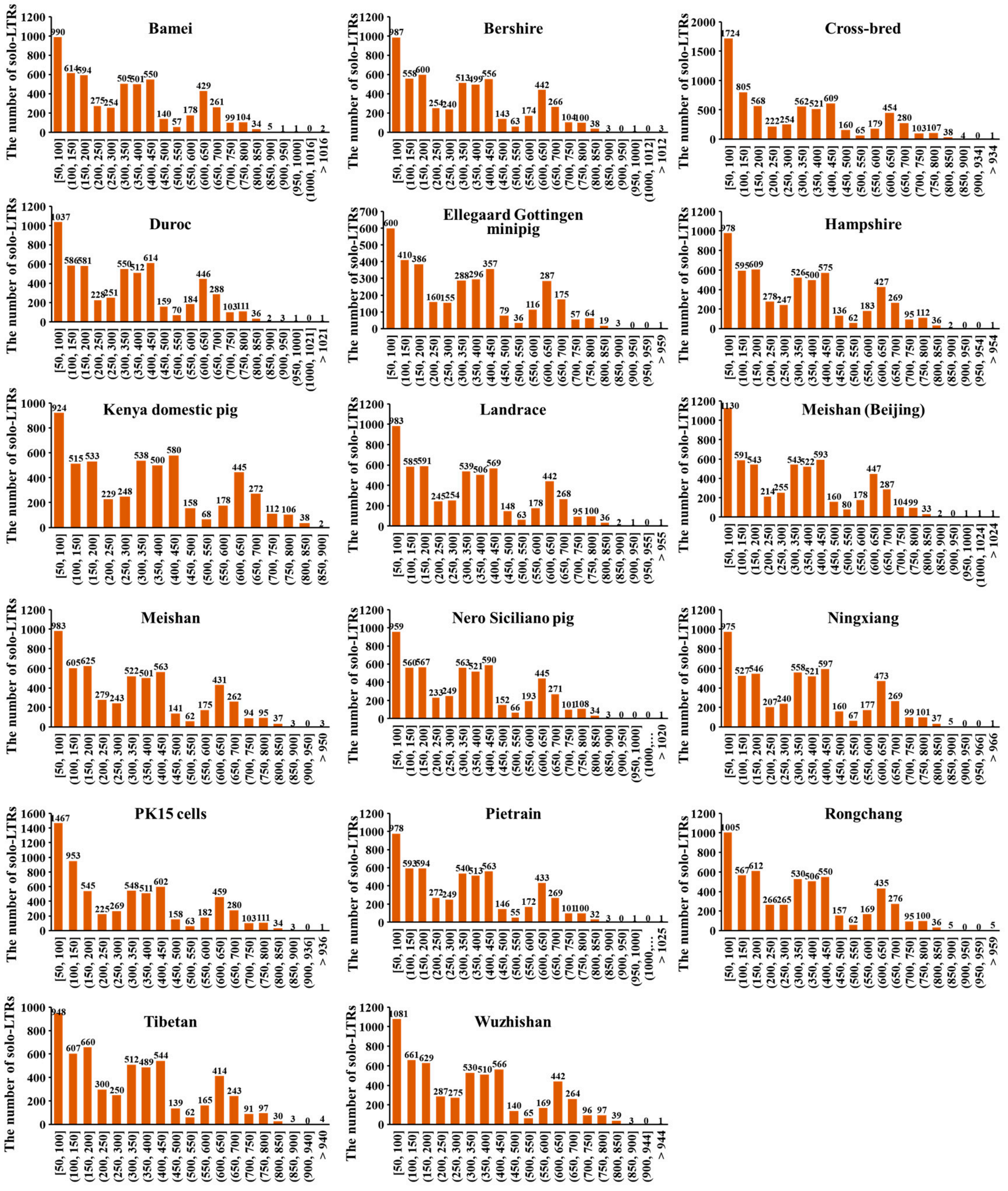

Figure S2. The length distribution of solo-LTRs in the left 17 pig genomes. The x-axis represents length intervals, and the y-axis represents the number of solo-LTRs.

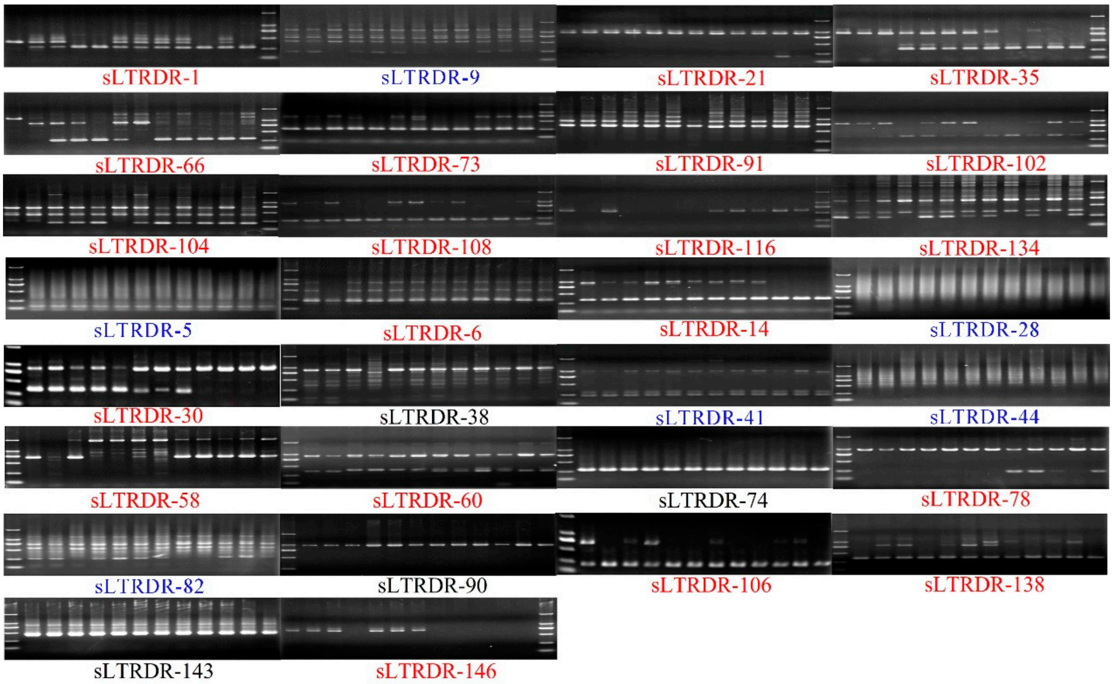

Figure S3. Gel electrophoresis results of 30 solo-LTR^{+} sites. For each lane, the larger band represent the solo-LTR was presence, while the small band represent the solo-LTR was absence. sLTRDR-38 and sLTRDR-90 are presence monomorphic, sLTRDR-74 and sLTRDR-143 are absence monomorphic, the names in red are polymorphic, names in black are monomorphic, name in blue are indeterminate. Lane order: Duroc, Large White, Landrace, Bamei, Ningxiang, Bama, Wuzhishan, Meishan, Mi, Sushan, Tibetan and Banna pigs. Marker: DL2000.

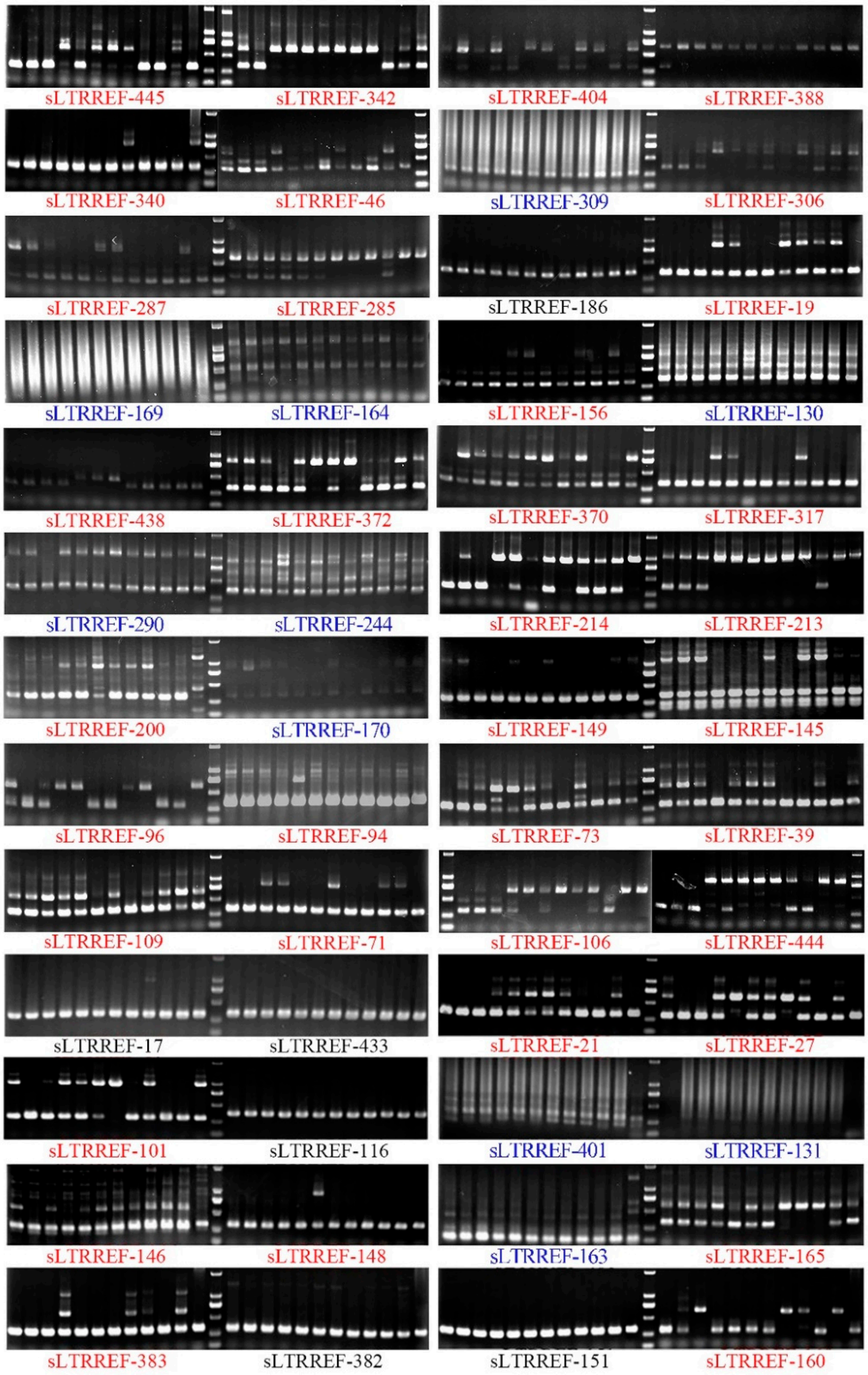

Figure S4. Gel electrophoresis results of 52 solo-LTR^{-} sites. For each lane, the larger band represent the solo-LTR was presence, while the small band represent the solo-LTR was absence. sLTRREF-17, sLTRREF-116, sLTRREF-151, sLTRREF-186, sLTRREF-382 and sLTRREF-433 are absence monomorphic, the names in red are polymorphic, names in black are monomorphic, name in blue are indeterminate. Lane order: Duroc, Large White, Landrace, Bamei, Ningxiang, Bama, Wuzhishan, Meishan, Mi, Sushan, Tibetan and Banna pigs. Marker: DL2000.

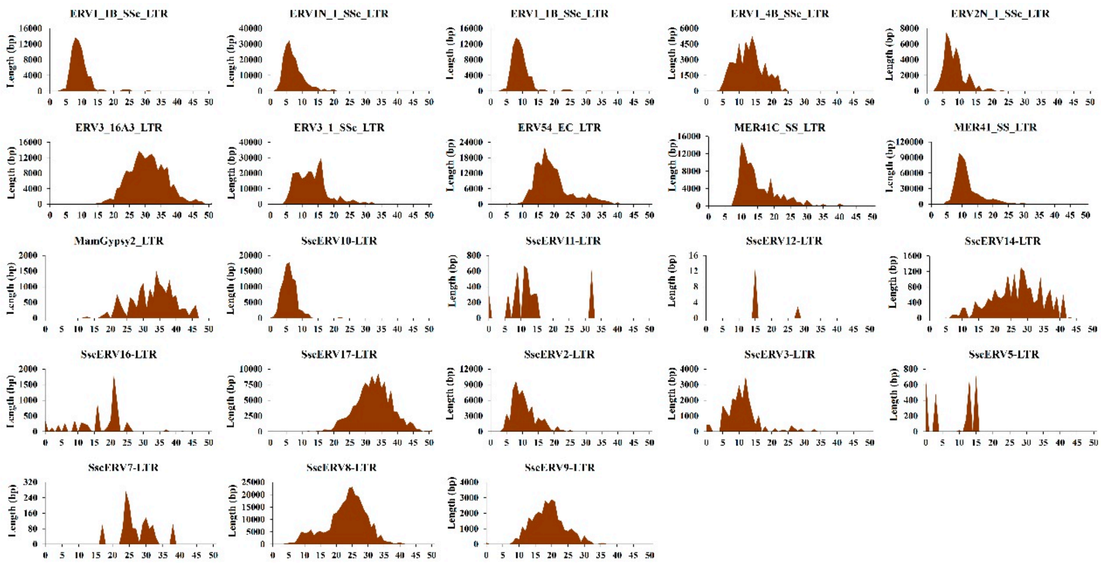

Figure S5. The divergence of LTR elements from 23 LTR retrotransposons within the pig genome are examined. The x-axis shows the divergence, while the y-axis represents the genomic content.

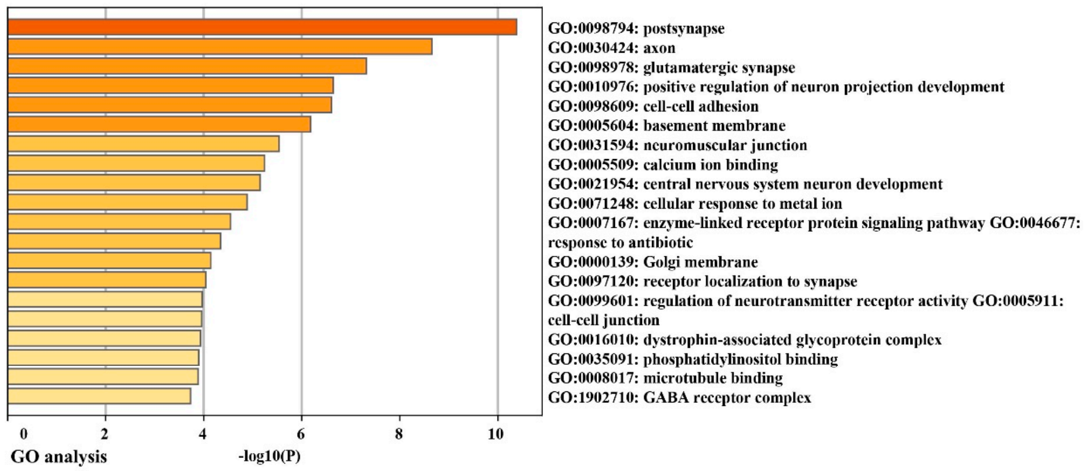

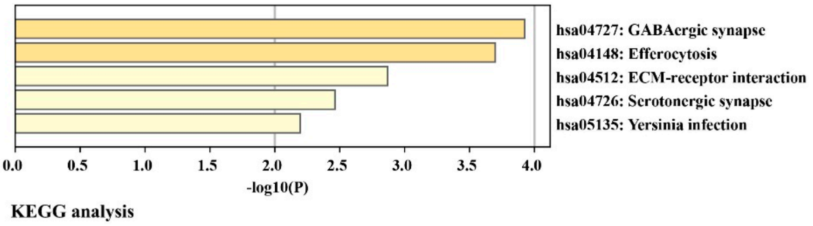

Figure S6. GO and KEGG analyses on protein-coding genes containing solo-LTR polymorphic sites. The above is GO analysis and the below is KEGG analysis.
